# Challenges in treating HIV-infected patient with disseminated Kaposi`s sarcoma and miliary TB

**DOI:** 10.1186/1471-2334-14-S4-P42

**Published:** 2014-05-29

**Authors:** Ana Maria Iancu, Irina Dumitru, Roxana Cernat, Sorin Rugină

**Affiliations:** 1 Clinical Hospital of Infectious Diseases, Constanța, Romania; 2 Ovidius University, Constanța, Romania

## 

Kaposi`s Sarcoma (KS) is the most common malignancy in HIV-infected patients. It`s induced by an infection with the Human Herpesvirus 8. AIDS-related Kaposi sarcoma tends to have an aggressive clinical course because it may involve all skin, membranes, lymph nodes, stomach, gut, lung or liver.

A 32-year-old patient was diagnosed in 2009 with HIV infection and disseminated KS. Purple macules and nodules were disseminated on the trunk, limbs and lingual mucosa. He was a late presenter with CD4-T cell count 30 cells/µL and 108,000 copies/mL HIV-RNA. With antiretroviral therapy (ART) the clinical, virological and immunological evolution were very good: the CD4-T cell count increased to 156 cells/µL and the viral load was undetectable. Unfortunately, he became non adherent, and after 2 years of absence he returned in very bad condition with pericarditis, pleurisy, ascites and a low CD4-T cell count (13 cells/µL). The changes showed by the chest CT scan, the results of pleural biopsy, the clinical findings raised the suspicion of disseminated KS and miliary tuberculosis. Despite all the treatments (Mega-ART, antituberculosis chemotherapy, opportunistic infections prophylaxis) the pericarditis and pleural fluid was always recovered. Weekly thoracentesis were needed and CD-4 T cell number had not increased as we expected (the maximal value was 88 cells/µL), although the viremia was undetectable.

There are 5 years since our patient was diagnosed with HIV infection and disseminated KS. In front of a patient with HIV-infection and symptomatic visceral or pulmonary KS, regardless the viral load, cytotoxic chemotherapy is recommended after the immunological status is improved. Another option is thoracoscopic talc pleurodesis which has been reported extremely effective in the malignant pleural effusion.

